# Cationic nanoparticles directly bind angiotensin-converting enzyme 2 and induce acute lung injury in mice

**DOI:** 10.1186/s12989-015-0080-x

**Published:** 2015-03-07

**Authors:** Yang Sun, Feng Guo, Zhen Zou, Chenggang Li, Xiaoxu Hong, Yan Zhao, Chenxuan Wang, Hongliang Wang, Haolin Liu, Peng Yang, Zongsheng Han, Kangtai Liu, Keiji Kuba, Bin Song, Jinming Gao, Ziyao Mo, Dangsheng Li, Bo Li, Qihan Li, Nanshan Zhong, Chen Wang, Josef M Penninger, Chengyu Jiang

**Affiliations:** State Key Laboratory of Medical Molecular Biology, Institute of Basic Medical Sciences, Chinese Academy of Medical Sciences, Peking Union Medical College, Tsinghua University, Beijing, 100005 China; Institute of Medical Biology, Chinese Academy of Medical Sciences, No. 379, Jiaoling Road, Kunming, Yunnan 650118 China; Chinese Pharmacopeia Commission, No. 11 Building Fahuananli Chongwen District, Beijing, 100060 China; National Center for Nanoscience and Technology, Beijing, 100190 China; IMBA, Institute of Molecular Biotechnology of the Austrian Academy of Sciences, Dr. Bohrgasse3, A-1030 Vienna, Austria; Center for Translational Medicine, Peking Union Medical College Hospital, Peking Union Medical College & Chinese Academy of Medical Sciences, Beijing, 100730 PR China; State Key Laboratory of Respiratory Diseases, Guangzhou Institute of Respiratory Diseases, The First Affiliated Hospital of Guangzhou Medical University, 151 Yanjiang Rd, Guangzhou, Guangdong 510120 China; Shanghai Institutes for Biological Sciences, Chinese Academy of Sciences, Shanghai, 200031 China; National Center for Safety Evaluation of Drugs, National Institutes for Food and Drug Control, Hongda Middle Street A8, Beijing Economic and Technological Development Area, Beijing, 100176 China; State Key Laboratory of Biotherapy/Collaborative Innovation Center for Biotherapy, West China Hospital, Sichuan University, Chengdu, 610000 China

**Keywords:** Nanoparticles, Angiotensin II, Angiotensin-converting enzyme 2, Acute lung injury, Losartan

## Abstract

**Background:**

Nanoparticles have become a key technology in multiple industries. However, there are growing reports of the toxicity of nanomaterials to humans. In particular, nanomaterials have been linked to lung diseases. The molecular mechanisms of nanoparticle toxicity are largely unexplored.

**Methods:**

Acute lung injury was induced in wild-type mice and angiotensin-coverting enzyme 2 (ACE2) knockout mice by the intratracheal instillation of cationic polyamidoamine dendrimer (PAMAM) nanoparticles. For rescue experiments, losartan (15 mg/kg in PBS) was injected intraperitoneally 30 min before nanoparticle administration.

**Results:**

Some PAMAM nanoparticles, but not anionic PAMAM nanoparticles or carbon nanotubes, triggered acute lung failure in mice. Mechanistically, cationic nanoparticles can directly bind ACE2, decrease its activity and down-regulate its expression level in lung tissue, resulting in deregulation of the renin-angiotensin system. Gene inactivation of *Ace2* can exacerbate lung injury. Importantly, the administration of losartan, which is an angiotensin II type I receptor antagonist, can ameliorate PAMAM nanoparticle-induced lung injury.

**Conclusions:**

Our data provide molecular insight into PAMAM nanoparticle-induced lung injury and suggest potential therapeutic and screening strategies to address the safety of nanomaterials.

**Electronic supplementary material:**

The online version of this article (doi:10.1186/s12989-015-0080-x) contains supplementary material, which is available to authorized users.

## Background

Nanoparticles have become key materials in the pharmaceutical, information, and communication industries as well as in multiple other areas that require strong, light materials [1]. However, several concerns have been raised about the safety of the widespread use of nanoparticles [2,3]. Among these concerns, the potential toxicity of nanoparticles to humans is among the most distressing. Nanomaterials have been reported to be harmful at the cellular, subcellular, and protein levels, and they have been found to elicit injurious responses in various organisms [4-6]. Many of these studies have focused on lung diseases, including clinical studies of workers who developed pulmonary disease from nanoparticle exposure [7-11]. A worldwide moratorium on nanomaterials has even been called until the safety issues have been resolved [12]. Nanoparticle safety in the respiratory system is important not only for workers who have high exposure to the particles but also in pulmonary drugs where nanoparticles serve as a vehicle directly to the lung [13]. Therefore, it is of critical importance to elucidate the molecular mechanisms by which nanoparticles induce lung injury.

Polyamidoamine (PAMAM) dendrimers are a family of dendritic polymers that are based on an ethylenediamine core and repeated amidoamine branching. As PAMAM dendrimers grow through generations 1–10 (G1-G10, G represents the generation of the dendrimers, which are based on an ethylenediamine core and repeated amidoamine branching), their size increases from 1.1–12.4 nm [14,15]. PAMAM dendrimers are commercially available as either whole (cationic) or half (anionic) generation polymers. The low polydispersity index and the ability to precisely control their surface chemistry make dendrimers useful for biomedicine applications such as drug or gene delivery and diagnostic imaging [6,14]. For example, PAMAM dendrimers can be used to inhibit inflammation [16], improve therapeutic responses against cancer [17], and suppress HIV-1 infection [18]. In addition, PAMAM dendrimers can also be used as nanocarriers for pulmonary drug delivery [19,20] or as an effective vector for pulmonary gene transfer [21]. Because many PAMAM nanoparticles have been considered carriers for pulmonary drug delivery, in addition, a study reported that the PAMAM can be found in major organs and have highest levels in lungs in B16 melanoma and DU145 human prostate cancer mouse tumor model [22], the safety of PAMAM nanoparticles should be under rigorous assessment and evaluation [23].

Previous reports have indicated that PAMAM dendrimers are toxic, and their toxicity is significantly influenced by size and surface charge [24]. Specifically, the charge of nanoparticles can also affect the oral toxicity of PAMAM dendrimers [25]. Furthermore, PAMAM dendrimers have been demonstrated to aggressively initiate blood clot formation and disrupt key platelet functions [26,27]. Although the systemic and oral toxicity of PAMAM nanoparticles has been investigated, there are few studies that have focused on the safety of PAMAM nanoparticles in the respiratory system. Considering the potential biomedical applications of PAMAM nanoparticles, especially the applications in pulmonary disease, it is paramount to investigate the underlying molecular mechanisms that may be involved in PAMAM-induced lung injury *in vivo*.

## Results

### G5 of PAMAM nanoparticles induce acute lung injury in mice

**Figure 1:**
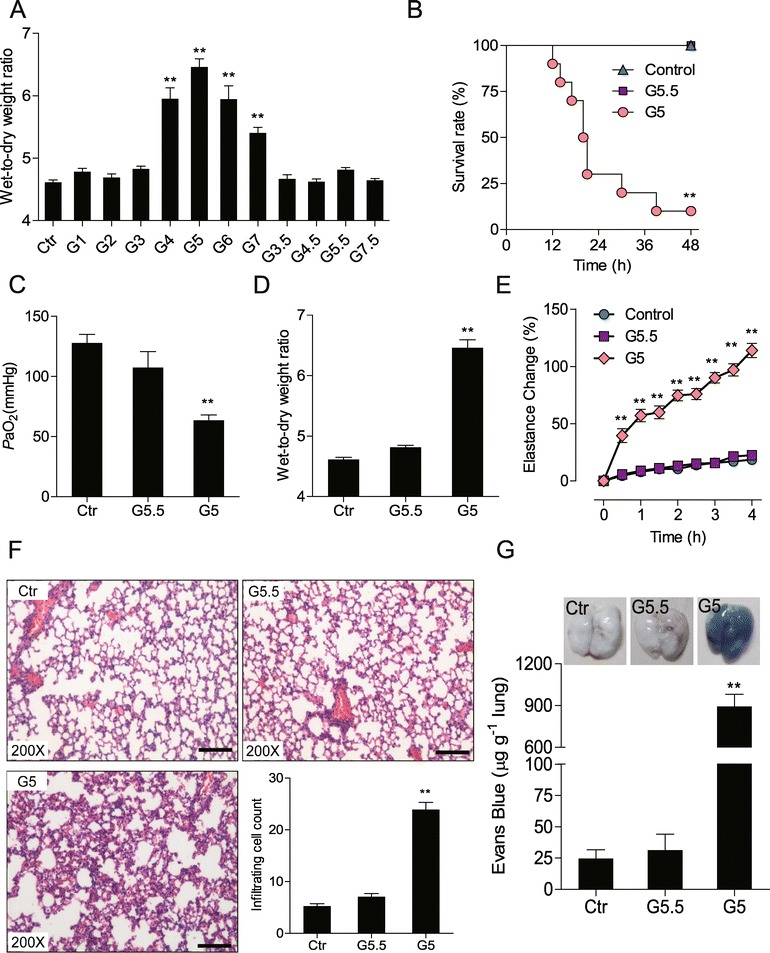
**G5 PAMAM dendrimers induce severe acute lung injury. (A)** Wet-to-dry weight ratios of the lungs after the administration of nanoparticles (15 μg/g) at 10 hrs after administration. n = 4–6 mice per group. **p < 0.01 compared with the G4-, G5-, G6-, and G7-treated groups and the vehicle control cohort using two-tailed *t*-test analysis. **(B)** Survival rates. n = 10 mice per group. **p < 0.01 for the comparison of the G5 group with either the G5.5 or control group. (log-rank test). **(C)** Arterial blood partial oxygen pressure (*P*aO_2_) and **(D)** Lung wet-to-dry weight ratios 10 hrs after the intratracheal instillation of the vehicle control, G5.5 (15 μg/g), or G5 (15 μg/g). n = 5 mice per group. **p < 0.01 for the comparison of the G5-treated cohorts with the G5.5-treated and control groups. (two-tailed *t*-test). **(E)** Change in lung elastance following a challenge with nanoparticles (15 μg/g) or the vehicle. n = 5–6 mice per group. (ANOVA with Bonferroni post-hoc analysis). **(F)** Representative images of lung pathology 10 hrs after the administration of the vehicle control or G5.5 or G5 nanoparticles (15 μg/g). The mean number of infiltrating cells per microscopic field ± SEM is also shown. n = 100 fields analyzed for three mice for each treatment group. (two-tailed *t*-test). Scale bar = 100 μm. **(G)** Representative images of lungs injected with Evans blue 10 hrs after challenge with nanoparticles (15 μg/g) or the vehicle (control). The amount of extravascular Evans blue was determined 10 hrs after the injection of nanoparticles or vehicle. n = 4–5 mice per group. (two-tailed *t*-test). Data are shown as the mean values ± SEM, except the survival curve. *p < 0.05 or **p < 0.01.

To determine whether PAMAMs can induce lung injury in mice*,* we tested different generations of PAMAMs via intratracheal administration (15 μg/g) approach *in vivo* (Detailed physical-chemical characteristic of PAMAMs used in this study were list in Additional file 1: Table S1). The G4, G5, G6, and G7 PAMAM dendrimers induced severe lung injury, as evidenced by an increased wet-to-dry weight ratio, which is a measure of lung edema and inflammation (Figure 1A). Among all of the tested nanoparticles, high generations of cationic PAMAM nanoparticles induced severe alterations in the lungs, whereas low generations of PAMAM and carboxyl-terminated dendrimers showed no acute toxicology; these results were consistent with previous studies [24,26-29]. Because G5 PAMAM nanoparticles (diameter is approximately 5 nm) exhibited the most severe acute lung injury, we focused on the G5 PAMAM nanoparticles in subsequent experiments. We also tested the acute toxicology of G5 PAMAM nanoparticles *in vivo*; the result showed that the LD50 of the G5 PAMAM nanoparticle was 11.22 μg/g and the 95% confidence limit was 9.89-12.74 μg/g (Table 1).

**Table 1:** **The result of acute toxicity of G5 PAMAM nanoparticles in mice**

**Group**	**Dose**	**Animal number**	**Number of animals**	**Mortality**	**Livability**
	**(μg/g)**	**(n)**	**dead/dosed**	**p**	**q**
I	0	6	0	0.00	1.00
II	7.35	6	1	0.17	0.83
III	10.5	6	2	0.33	0.67
IV	15	6	5	0.83	0.17
V	21.43	6	6	1.00	0.00
VI	30.61	6	6	1.00	0.00

The percent mortality was recorded at 48 hrs after treatment with G5 PAMAM nanoparticles.

We first examined the survival rate of mice after the administration of G5 PAMAM dendrimers to the lungs. Almost all mice treated with G5 PAMAM nanoparticle died within 48 hrs, whereas all control mice and mice instilled with anionic G5.5 PAMAM dendrimers survived (Figure 1B). The cause of death was rapid development of severe lung injury characterized by decreased blood oxygenation (Figure 1C), increased lung tissue wet-to-dry weight ratios (Figure 1D), and massively altered elastance (Figure 1E). (Lung elastance is a measure of lung function, i.e., the change in pressure per unit change in volume, and indicates the stiffness of the lungs [30].) The histopathology of G5 PAMAM-treated lungs showed increased bleeding and inflammatory cell infiltration (Figure 1F). Moreover, G5 PAMAM administration resulted in increased vascular permeability (Figure 1G), another hallmark of acute lung injury/respiratory distress syndrome in humans [31,32]. These results indicate that the inhalation of G5 PAMAM nanoparticles induces acute lung injury in mice, resulting in pathologies that resemble acute respiratory distress syndrome in humans.

### ACE2 controls G5 PAMAM nanoparticle-induced lung injury

**Figure 2:**
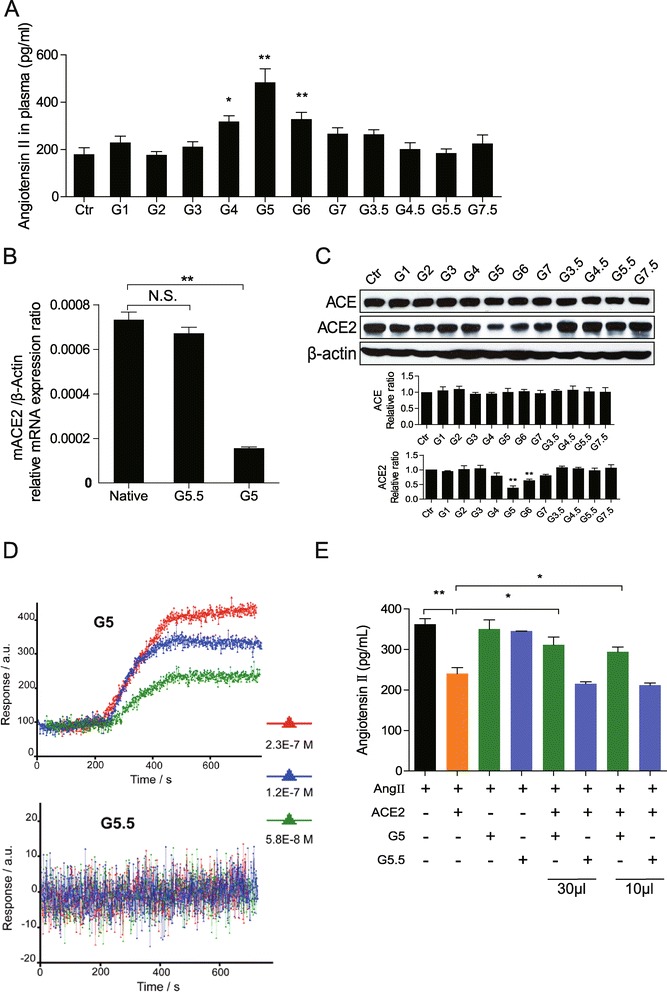
**Down-regulated ACE2 expression in mice challenged with cationic PAMAM dendrimers. (A)** Levels of AngII in the plasma of the vehicle- (control) and nanoparticle-treated (15 μg/g) mice at 3 hrs after administration. AngII levels were determined using radioimmunoassays. n = 4–5 mice per group. *p < 0.05 or **p < 0.01 for the comparison of the G4, G5-, and G6-treated groups with the vehicle (control) group (two-tailed *t*-test). **(B)** The ACE2 mRNA relative expression level of the vehicle- (control) and nanoparticle-treated (15 μg/g) mice at 3 hrs after administration. Data were normalized to the expression of β-actin reference gene. (two-tailed *t*-test). **(C)** Western blots of total lung samples obtained 3 hrs after the instillation of nanoparticles (15 μg/g). The blots are representative of three different mice for each treatment. Quantitative analyses of the ACE and ACE2 protein levels are illustrated. The levels are shown as the mean ACE- and ACE2-to-β-actin ratios ± SEM. n = 3 mice per treatment. **p < 0.01 for the comparison of the G5-, and G6-treated groups with the vehicle group (two-tailed *t*-test). **(D)** Binding of G5 and G5.5 nanoparticles to recombinant human ACE2 at different concentrations was measured by surface plasmon resonance (SPR). The detailed dynamic binding constant and equilibrium dissociation constant are shown in Table S2. **(E)** G5 and G5.5 nanoparticles at different concentrations were incubated with recombinant ACE2 and AngII. The levels of AngII in the enzymatic activity measurement system were determined by radioimmunoassay. n = 3 tests per group. (two-tailed t-tests). Data are shown as the mean values ± SEM. *p < 0.05 or **p < 0.01; N.S. means not significant.

We previously discovered a role for ACE2 in protecting mice from severe acute lung injury [33-35]. ACE2 proteolytically cleaves angiotensin II (AngII) and thereby negatively regulates the renin-angiotensin system (RAS) [36]. The induction of acute lung injury by acid aspiration, sepsis, or SARS-CoV resulted in the down-regulation of ACE2 expression in mouse lung tissue and the subsequent upregulation of AngII [33,34]. We also reported that plasma AngII levels are linked to disease severity and can predict fatal outcomes in H7N9-infected patients [37]. We therefore tested whether the RAS was also affected during nanoparticle-induced lung injury in mice. The serum AngII levels were measured in mice following the inhalation of PAMAMs, and other nanomaterials (Figure 2A, Additional file 2: Figure S1A). Additionally, ACE2 mRNA (Figure 2B) and protein levels in the lungs were determined (Figure 2C, Additional file 2: Figure S1B). The RAS system was markedly activated, as demonstrated by the increased serum AngII levels in mice treated with G4, G5, and G6 cationic PAMAM nanoparticles; the G5 PAMAM particles had the greatest effect (Figure 2A). Moreover, the inhalation of G5 and G6 PAMAM dendrimers resulted in significant down-regulation of ACE2 protein expression in lung tissue, again with G5 PAMAM inducing the greatest effect (Figure 2C). Inhalation of G4 and G7 PAMAM nanoparticles also resulted in reduced ACE2 expression in the lungs, although these differences did not reach statistical significance. Notably, the lung levels of ACE, the enzyme that generates AngII [38], were apparently not affected by PAMAM inhalation (Figure 2C). Thus, the pulmonary administration of certain PAMAM nanoparticles, especially G5 PAMAM, markedly activate the RAS via the down-regulation of ACE2 expression.

To further elucidate the mechanism of ACE2 down-regulation, we first explored whether the loss of ACE2 activity was caused by the direct binding of ACE2 to nanoparticles. We assessed the binding affinity of ACE2 for various nanoparticles using surface plasmon resonance (SPR). Interestingly, most cationic PAMAMs could bind ACE2 protein directly, whereas the anionic PAMAMs failed to bind ACE2 (Figure 2D, Additional file 3: Figure S2). This is predictable since the theoretical isoelectric point of protein ACE2 calculated by Expasy (http://www.expasy.org) is 5.36 and the SPR assay is performed in neutral pH. The dynamic binding constants and equilibrium dissociation constants for the binding between the examined nanoparticles and ACE2 were also measured, demonstrating that G5 PAMAM nanoparticles, in particular, strongly bind to ACE2 (Additional file 4: Table S2). We next monitored ACE2 enzyme activity by measuring AngII levels after mixing recombinant ACE2 protein and various nanoparticles *in vitro*. The enzymatic activity of ACE2 was decreased after incubation with cationic G5 PAMAM nanoparticles, but its activity was not affected after incubation with anionic G5.5 PAMAM *in vitro* (Figure 2E). These data show that G5 PAMAM nanoparticles can directly associate with ACE2 and alter its enzymatic activity, thereby resulting in activation of the RAS.

**Figure 3:**
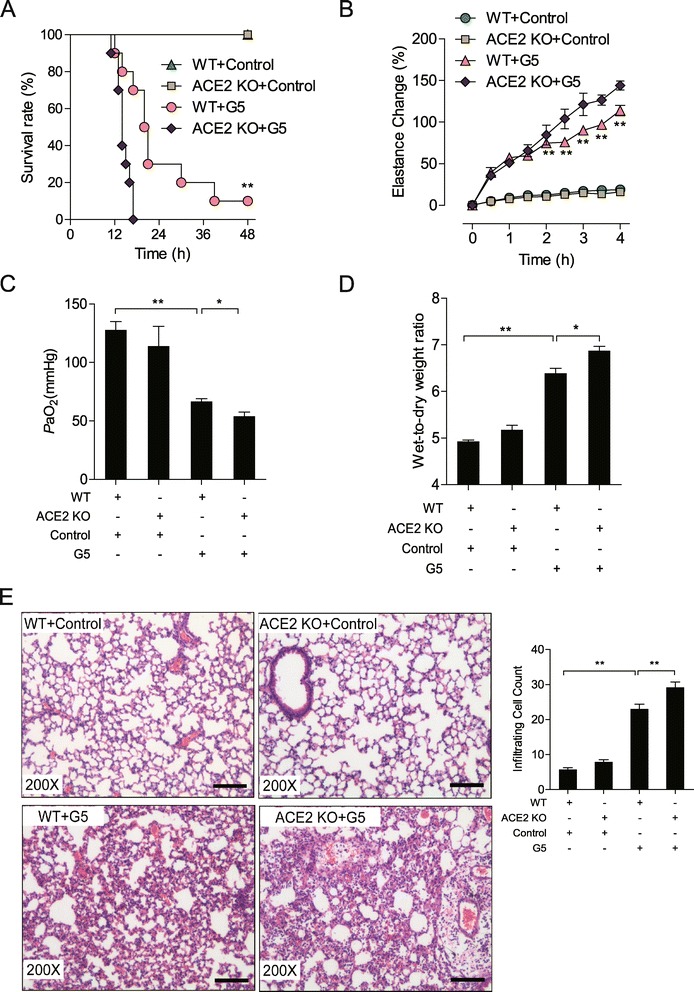
***Ace2*** **deficiency increases the severity of G5 PAMAM nanoparticle-induced acute lung injury. (A)** Survival rates of vehicle- (control) or G5 PAMAM-treated (15 μg/g) wild-type (WT) and *Ace2*-knockout (ACE2 KO) mice. n = 10 mice per group. **p < 0.01 for the comparison of the WT + G5 group with the ACE2 KO + G5 group (log-rank test). **(B)** Percent changes in the lung elastance of the vehicle control and PAMAM G5-treated (15 μg/g) WT and ACE2 KO mice at the indicated time points. n = 6 mice per group. **p < 0.01 for the comparison of the WT + G5 group with the ACE2 KO + G5 group at the indicated time points. (ANOVA with Bonferroni post-hoc analysis). **(C)** *P*aO_2_ in the arterial blood of vehicle- (control) or G5 PAMAM-treated (15 μg/g) WT and ACE2 KO mice. n = 4–7 mice per group. (two-tailed *t*-test). **(D)** Wet-to-dry weight ratios of the lungs of WT and ACE2 KO mice 10 hrs after intratracheal instillation of vehicle (control) or G5 PAMAM (15 μg/g). n = 4 mice per group. (two-tailed *t*-test). **(E)** Representative lung pathologies of WT and ACE2 KO mice 10 hrs after the administration of vehicle (control) or G5 PAMAM (15 μg/g). The numbers of infiltrating cells per microscopic field ± SEM are also shown. n = 100 fields analyzed for three mice for each treatment. (two-tailed *t*-test). Scale bar = 100 μm. Data are shown as the mean values ± SEM, except the survival curve. *p < 0.05 or **p < 0.01.

Finally, to determine whether ACE2 has a critical role in the acute lung injury following G5 PAMAM dendrimer administration, we examined mice that were genetically deficient in *Ace2* [36]. Following the administration of G5 PAMAM dendrimers, the *Ace2-*knockout mice died significantly faster than the wild-type control mice (Figure 3A). In addition, the G5 PAMAM-induced changes in lung elastance, blood oxygenation, lung tissue wet-to-dry weight ratio, and lung histopathology were exacerbated in *Ace2*-knockout mice relative to *Ace2*-expressing control animals (Figures 3B-E). These data indicate that G5 PAMAM dendrimers can induce severe acute lung failure by functionally altering the RAS, specifically via the down-regulation of ACE2.

### Losartan alleviates G5 PAMAM nanoparticle-induced lung injury in mice

**Figure 4:**
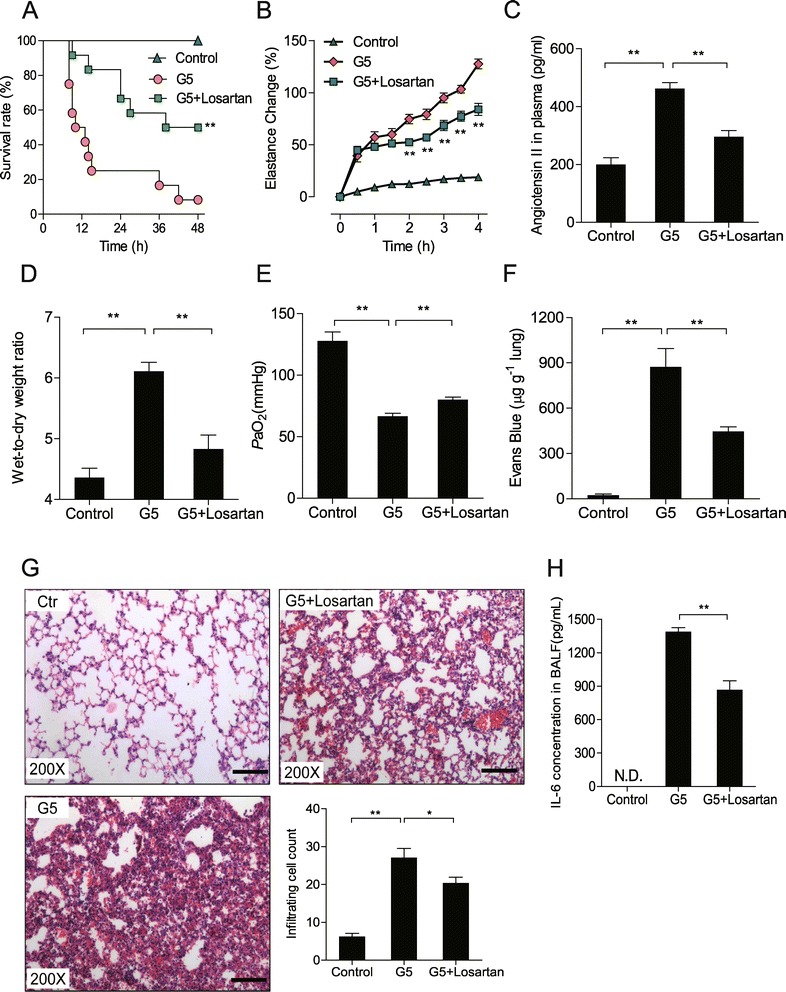
**Losartan reduces the severity of G5 PAMAM nanoparticle-induced acute lung injury. (A)** Survival rates at indicated times (log-rank test), **(B)** percent change in lung elastance at indicated times (ANOVA with Bonferroni post hoc analyses), **(C)** plasma AngII levels (at 3 hrs, two-tailed t-tests), **(D)** lung wet-to-dry weight ratios (at 10 hrs, two-tailed t-tests), **(E)** blood oxygenation (at 10 hrs, two-tailed t-tests), **(F)** vascular leakage (at 10 hrs, two-tailed t-tests), **(G)** histopathology, lung infiltrating cells counting (at 10 hrs) and **(H)** IL-6 concentration in BALF (at 10 hrs) of control WT mice, WT mice treated with G5 PAMAM nanoparticles (15 μg/g), and WT mice treated with G5 PAMAM nanoparticles (15 μg/g) plus losartan (15 mg/kg i.p.). n = 4–10 mice per group. (two-tailed t-tests). In **(G)**, representative lung histopathologies and the mean numbers of lung-infiltrating cells ± SEM per microscopic field (100 fields were analyzed; n = 3 mice per group) are shown. Scale bar = 100 μm. Data are shown as the mean values ± SEM, except the survival curve. *p < 0.05 or **p < 0.01. N.D. means not detectable.

We next tested whether losartan, the first angiotensin receptor blocker (ARB) approved for clinical use, could rescue the severe lung injury that occurred following the inhalation of G5 PAMAM dendrimers. Importantly, the administration of losartan prolonged the overall survival time of G5 PAMAM-treated wild type mice (Figure 4A), markedly alleviated their impaired lung function (Figure 4B), attenuated their increased AngII production (Figure 4C), reduced the increased wet-to-dry weight ratio of their lung tissue (Figure 4D), and improved the decreased blood oxygenation (Figure 4E). As measured by Evans blue vascular leakage assays, losartan also significantly decreased the vascular permeability of the pulmonary blood vessels in G5 PAMAM-treated mice (Figure 4F). In addition, treatment with losartan ameliorated G5 PAMAM-induced lung histopathology, including bleeding and inflammation cell infiltration (Figure 4G). Furthermore, we measured interleukin-6 (IL-6), which was a classical pro-inflammatory cytokine, in bronchoalveolar lavage fluid (BALF). Enzyme-linked immuno sorbent assay (ELISA) result showed treatment with losartan could reduce concentration of IL-6 in BALF after instillation of G5 PAMAM dendrimers. (Figure 4H). Thus, nanoparticle-induced lung injury can be inhibited by the administration of losartan.

## Discussion

Since G5 PAMAM dendrimers can down-regulate the expression of ACE2 in mRNA and protein levels significantly after 3 hrs of instillation (Figure 2 B,C), and death of mice occurred about 12 hours after instillation of PAMAM G5 (Figure 1B), we choose time-point 10 hrs after instillation of PAMAMs to evaluate the change of lung function. Our data provide *in vivo* molecular insight into the acute lung failure that can be induced by nanomaterials. Our data indicate that G5 PAMAM dendrimers down-regulate ACE2 expression in lung tissue, resulting in increased AngII production and, consequently, acute lung failure. Our SPR data show that PAMAM G5 and protein ACE2 have the highest binding affinity (Additional file 4: Table S2), however, PAMAM G7 have the most amino surface groups (Additional file 1: Table S1), suggesting that the nanoparticles surface charge is not the only determinant. Further studies of conformation characteristic and stability of PAMAM G5-ACE2 complex are needed.

In our study, PAMAM nanoparticles of G4-G7 could induce lung edema (Figure 1A), whereas lower generation cationic and anionic PAMAM dendrimers exhibited no acute lung toxicity. This result is consistent with previous findings that state nanoparticle size significantly influences the toxicology of PAMAM nanoparticles [24]. It is interesting to note that not all nanoparticles have such effects on the ACE2 and AngII levels; for example, G5.5 PAMAM dendrimers appear to be less toxic than G5 PAMAM dendrimers. Additional studies regarding the entry of nanoparticles into pulmonary epithelial cells and/or local resident macrophages are required to explain these differences. Nevertheless, minor modifications of nanomaterials appear to significantly reduce their pulmonary toxicity, suggesting a possible approach to enable a safer use of nanomaterials. We have previously shown that G3-8 cationic PAMAM nanoparticles, COOH single-walled carbon nanotube, and copper oxide nanoparticles induce autophagy *in vitro* in A549 cell models or *in vivo* in mouse models [9,39,40]. Thus, the severity of the lung injury triggered by PAMAM nanoparticles may be due to the activation of multiple signaling pathways that collectively cause severe lung disease. A previous study showed that AngII could increase autophagy levels via activation of the AT1 receptor and NADPH oxidase in vascular muscle cells [41]; whether this signaling transduction pathway is involved in the pathogenesis of lung failure induced by G5 PAMAM nanoparticles needs further study. Our new data now clearly establish that ACE2 and the RAS play key roles in G5 PAMAM nanoparticle-induced lung injury. Interestingly, these mechanisms also apply in severe acute lung injury associated with acid aspiration, sepsis, or infection with SARS-CoV, H5N1 and H7N9 influenza [33-35,42]. Thus, these parallel mechanisms suggest that ACE2 and RAS share a common disease mechanism for acute lung injury and respiratory distress syndrome. Modulating the RAS may have a wide range of therapeutic applications.

The development of nanotechnology has rapidly accelerated, with an estimated market of approximately one trillion US dollars within the next few years [43]. Safety problems associated with nanomaterials have recently attracted attention, highlighting the urgent need for protocols that protect workers, consumers, and the environment [44]. We propose here a valuable strategy using ARB drug to address potential acute lung injury might be induced by application of PAMAMs.

## Conclusions

We show that cationic PAMAM dendrimer nanoparticles can trigger acute lung failure in mice. Mechanistically, cationic nanoparticles bind to ACE2 and down-regulate its function and expression in lung tissue, resulting in deregulation of the RAS. Importantly, the administration of losartan ameliorated the nanoparticle-induced lung injury.

Our new insight into the molecular pathogenesis of cationic PAMAM nanoparticle-induced lung failure might therefore contribute to a better understanding of nanoparticle toxicity and the development of improved safety procedures for nanomaterials. Because ARB drugs have a long historical application for hypertension, our results not only uncover an important pathway of cationic PAMAM nanoparticle-induced lung toxicity, but may allow translation of our findings to novel therapies using classical ARB drugs.

## Methods

### Reagents

All PAMAM dendrimers, carbon nanotubes, and inorganic nanoparticle powders were purchased from Sigma-Aldrich PAMAM nanoparticles were air-dried on a super-clean bench for 24 hrs to remove the methanol before an equal volume of PBS was added to the tube. The samples were mixed and stored at 4°C before use. The anti-ACE antibody (2E2) was purchased from Santa Cruz Biotechnology, Inc. The anti-ACE2 antibody (clone 460502) and recombinant human ACE2 protein were purchased from R&D systems. The anti-β-actin antibody (clone AC-15), angiotensin II (human), losartan and Evans blue were purchased from Sigma-Aldrich. HS-PEG-COOH (Mw = 2000 Da), HS-PEG-NH2 (Mw = 2000 Da), and HS-(CH2)11-EG6-OH (Mw = 468 Da) were purchased from Shanghai Yare Biotechnology, Inc. 1-(3-Dimethylaminopropyl)-3-ethylcarbodiimide hydrochloride (EDC), *N*-hydroxysuccinimide (NHS), ethanol, NaOH, and ethanolamine were purchased from Sigma-Aldrich. All these reagents were used without further purification. Milli-Q water was obtained from a Millipore − ELIX water purification system. All buffers and reagents used were degassed and filtered prior to use in the SPR experiments.

### Animals

All mice had a C57BL/6 J background. The development of the *Ace2*-knockout mouse has been previously described [36]. C57BL/6 J mice were purchased from Vital River, Beijing. All mice were 4–6 weeks old. All animal experiments were conducted in the animal facility at the Institute of Basic Medical Sciences of the Peking Union Medical College in accordance with local and institutional guidelines.

### Induction of lung injury

Lung injury was induced in mice by the intratracheal instillation of nanoparticles at a concentration of 15 μg/g using a technology that we have previously described for SARS spike protein- and acid-induced lung injury [34]. For rescue experiments, losartan (15 mg/kg in PBS) was injected intraperitoneally 30 min before nanoparticle administration.

### Acute toxicity study

A total of 48 male mice were divided into six groups of six mice each and randomly allotted to the control and G5 PAMAM nanoparticle groups. PBS was used in Group I as a control. The mice in Groups II, III, IV, V and VI were intratracheally injected with G5 PAMAM nanoparticles with the concentration of 7.35, 10.5, 15, 21.43, 30.61 μg/g, respectively. The animals were observed for 48 hrs after administration with nanoparticles and the mortality rate was recorded. The LD50 value (95% CL) was calculated using the improved Karber's method. The LD50 was 11.22 μg/g and the 95% confidence limit was 9.89-12.74 μg/g.

### Assessment of pulmonary edema

Mice were instilled intratracheally with nanoparticles at a concentration of 15 μg/g before assessing the extent of pulmonary edema 10 hrs later when the lung wet-to-dry weight ratios were calculated. In brief, after the blood was drained from the excised lungs, the wet weight of the lungs was measured. Lungs were then heated to 68°C in an oven for 24 hrs and weighed to determine the baseline dry mass of the lungs.

### Survival rates

Mice were intratracheally injected with either the vehicle or nanoparticles (15 μg/g). The survival of each group was recorded for 48 hrs and plotted using Kaplan-Meier survival curves. In the rescue group, losartan was intraperitoneally injected 30 min before nanoparticle administration.

### Assessment of blood oxygenation

Mice were intratracheally injected with nanoparticles at a concentration of 15 μg/g. Ten hours later, blood samples were obtained from the common carotid artery, and the *P*aO_2_ was measured (Radiometer ABL720 blood gas analyzer, Diamond Diagnostics-USA) to assess arterial blood oxygenation as an indicator of respiratory failure.

### Lung elastance

Mice were anesthetized with sodium pentobarbital, and then either the vehicle (control) or nanoparticles (15 μg/g) dissolved in the same vehicle were administered intratracheally. Lung elastance was then measured by BUXCO Pulmonary Function Testing (PFT) every 30 min during spontaneous breathing periods for 4 hrs. In the rescue group, losartan (15 mg/kg) was intraperitoneally injected 30 min before nanoparticle administration.

### Histopathology

Ten hours after the administration of nanoparticles (15 μg/g), mice were sacrificed and their body weights determined. The lungs were removed from the thoracic cavity and placed in a glass vial containing approximately 50 mL of fixative. Each glass vial was assigned a number unknown to the pathologists. The lungs were fixed for at least 48 hrs in formalin, embedded in paraffin, thin-sectioned coronally, and mounted on glass slides using standard histopathological techniques. The sections were stained with hematoxylin and eosin. For each mouse, lung sections were examined independently by three pathologists.

### Pulmonary vascular permeability

Following the induction of lung injury, pulmonary vascular permeability was assessed by measuring the pulmonary extravasation of Evans blue. Evans blue (20 μg/g) was injected into the tail vein 10 hrs after the intratracheal injection of nanoparticles. Ten minutes later, the animals were sacrificed and their lungs were removed from the thoracic cavity and perfused with ice-cold PBS via injection into the right ventricle. Then, the lung tissue was immersed in formamide and analyzed to determine the Evans blue content. The OD value of the Evans blue formamide solution was read using a microplate spectrophotometer at 620 nm. A standard concentration regression curve was created and used to determine the level of Evans blue in the lung tissue.

### Angiotensin II peptide levels

Mice were intratracheally injected with nanoparticles (15 μg/g) and blood was collected 3 hrs later into 1.5 mL tubes containing a mixture of peptidase inhibitors (0.30 M ethylenediaminetetraacetic acid, 0.32 M dimercaprol, and 0.34 M 8-hydroxyquinoline sulfate). Radioimmunoassays to determine the levels of angiotensin peptide in the plasma extracts were performed as described previously [45]. In the rescue group, losartan (15 mg/kg) was intraperitoneally injected 30 min before nanoparticle administration.

### Real-time quantitative PCR analysis

For real-time quantitative PCR, lung tissue was obtained 3 hrs after the intratracheal injection of the nanoparticles (15 μg/g) and then homogenized using TRIzol reagent (Invitrogen). The RNA was extracted according to manufacturer’s instructions. Complementary DNA (cDNA) was synthesized from 1.5 μg of total RNA with the High Capacity cDNA Reverse Transcription Kit (Applied Biosystems). PCR amplification assays were performed with the LightCycler 480 SYBR Green I Master (Roche Applied Science) on a LightCycler 480 PCR System (Roche Applied Science). PCR products were confirmed by sequencing. Lung tissue samples were normalized to β-actin levels. The specific primers used were listed as follows:

mouse β-actin forward primer: 5'- CTCTCCCTCACGCCATCC-3'

mouse β-actin reverse primer: 5'- CGCACGATTTCCCTCTCAG-3'

mACE2 forward primer: 5'- GGCTACAACTATAACCGTAAC-3'

mACE2 reverse primer: 5'- TATCCATCAACTTCCTCCTC-3'.

### Western blotting

Lung samples were obtained 3 hrs after the intratracheal injection of the nanoparticles (15 μg/g) and homogenized in ice-cold lysis buffer (50 mM Tris–HCl, pH 7.5, 150 mM NaCl, 1.0% Triton X-100, 20 mM EDTA, 1 mM Na_3_VO_4_, 1 mM NaF, and protease inhibitors). Tissue lysates were separated by SDS-PAGE and proteins were transferred to NC membranes. The membranes were incubated with antibodies to ACE, ACE2, and β-actin, followed by incubation with a horseradish peroxidase–conjugated secondary antibody. The band intensity was analyzed using Quantity One software.

### Surface plasmon resonance (SPR) experiments

To immobilize analytes with an amino terminus, HS-PEG-COOH and HS-(CH2)11-EG6-OH (molar ratio, 1:10) at a total concentration of 1 mM were printed on a bare gold SPRi chip. To immobilize analytes with a carboxyl terminus, HS-PEG-NH2 and HS-(CH2)11-EG6-OH (molar ratio, 1:10) were printed on a bare gold SPRi chip at a total concentration of 1 mM. Chips were incubated in wet air at −4°C for 12 hrs to allow the formation of a uniform self-assembled mono-layer. Chips were washed with deionized water and ethanol and dried in a stream of N_2_. After activation with EDC/NHS for 10 min (0.1 M NHS, 0.4 M EDC), 1 mg/mL analyte (G1, G2, G3, G4, G5, G6, G7) was printed on the chip’s surface and the chips were incubated for 2 hrs at −4°C. The un-reacted esters were deactivated by exposing the chips to 1 M ethanolamine aqueous solution (pH = 8.5) for 10 min. For carboxyl terminal analytes, the unreacted area of the SPRi chip’s surface was deactivated by exposure to 1 M ethanolamine aqueous solution (including EDC/NHS solution) for 30 min. The surface was washed with deionized water and ethanol and then dried with nitrogen.

Real-time detection of protein adsorption was performed using a Plexera Κχ5 V2 SPRi apparatus (Plexera Bioscience LLC, USA). The entire SPRi chip surface was imaged during the angular scan. The immobilized analyte spots with 100 μm diameters on the SPRi chip were selected. The working optical position was automatically calculated according to the plasma curve. The auto-injection system controlled the flow at a rate of 2 μL⋅s^−1^. PBS (pH = 7.4) was used as the running buffer. When baseline was obtained, proteins at different concentrations (0.03 μM, 0.06 μM, 0.12 μM, and 0.23 μM) were injected into the flow cell. NaOH (20 mM) was used as the regeneration solution. In the kinetic analysis, the Plexera evaluation software was used to fit the SPR curves to the Langmuir equation.

### Enzymatic activity assays

Recombinant human ACE2 protein (20 ng/mL) was incubated with or without 8 mg/mL different nanoparticles (10 μL or 30 μL) at 37°C in assay buffer (75 mM Tris, 1 M NaCl, pH = 7.5) for 10 min before adding angiotensin II (400 pg/mL) for an additional 30 min at 37°C, except for the blank control sample. Radioimmunoassays to determine the level of angiotensin II peptide in the assay buffer were performed as described previously [45].

### Cytokine measurement

Bronchoalveolar lavage fluid (BALF) was obtained 10 hrs after the intratracheal injection of the nanoparticles (15 μg/g). The concentration of IL-6 in the BALF was determined by ELISA (R&D systems, DY406) according to the manufacturer’s instructions. In the rescue group, losartan (15 mg/kg) was intraperitoneally injected 30 min before nanoparticle administration.

### Statistical analyses

All data are shown as the mean values ± SEM. Measurements at single time points were analyzed by ANOVA, and if they demonstrated significance, they were further analyzed using a two-tailed *t*-test. Time courses were analyzed using repeated measures (mixed model) ANOVA with Bonferroni post-hoc analyses. All statistical tests were conducted using GraphPad Prism 5.0 (GraphPad Software, San Diego, CA, USA). A value of *p* < 0.05 was used to indicate statistical significance.

## Additional files

Additional file 1: Table S1. Physical-chemical characteristics of PAMAMs used in this study. Additional file 2: Figure S1. Effects of various nanomaterials on ACE2 expression and plasma angiotensin II levels. (A)Plasma AngII levels of mice receiving the vehicle (control) and mice instilled with the indicated nanoparticles (all used at15 μg/g) at 3 hrs after challenge. n = 4 mice per group. (B) ACE and ACE2 protein levels in lungs 3 hrs after the instillation of t he indicated nanoparticles (15 μg/g). The upper panels show representative Western blots. The bar graphs show ACE and ACE2 levels as ratios to β-actin. n = 3 mice per group. Data are shown as the mean values ± SEM. For A and B, no differences were detected between the nanoparticle-treated groups and the vehicle control groups using two-tailed t-tests. Additional file 3: Figure S2. SPR signal of cationic polyamidoamine dendrimer nanoparticles combined with ACE2. The binding abilities of G2, G3, G4, G6 and G7 nanoparticles with recombinant ACE2 at different concentrations were measured by surface plasmon resonance (SPR). The detailed dynamic binding constants and equilibrium dissociation constants are shown in Additional file 1: Table S1. Additional file 4: Table S2. Kinetic parameters for the binding between cationic polyamidoamine dendrimer nanoparticles and ACE2. Binding of cationic polyamidoamine dendrimer nanoparticles, including the cationic polymers G1, G2, G3, G4, G5, G6, and G7 and the anionicpolymers G3.5, G4.5, G5.5, and G7.5, to recombinant ACE2 was assessed using SPR. The detailed data for the ka (dynamic binding constant), kd (dynamic dissociation constant), KA (equilibrium binding constant) and KD (equilibrium dissociation constant) are indicated. N.D. indicates that no SPR signal was detected.

## References

[1] Brumfiel G (2006). Consumer products leap aboard the nano bandwagon. Nature.

[2] Yokel RA, Macphail RC (2011). Engineered nanomaterials: exposures, hazards, and risk prevention. J Occup Med Toxicol.

[3] Oberdorster G (2010). Safety assessment for nanotechnology and nanomedicine: concepts of nanotoxicology. J Intern Med.

[4] Nel A, Xia T, Madler L, Li N (2006). Toxic potential of materials at the nanolevel. Science.

[5] Ai J, Biazar E, Jafarpour M, Montazeri M, Majdi A, Aminifard S (2011). Nanotoxicology and nanoparticle safety in biomedical designs. Int J Nanomedicine.

[6] Pryor JB, Harper BJ, Harper SL (2014). Comparative toxicological assessment of PAMAM and thiophosphoryl dendrimers using embryonic zebrafish. Int J Nanomedicine.

[7] Inoue K, Takano H, Yanagisawa R, Hirano S, Kobayashi T, Fujitani Y (2007). Effects of inhaled nanoparticles on acute lung injury induced by lipopolysaccharide in mice. Toxicology.

[8] Kemp SJ, Thorley AJ, Gorelik J, Seckl MJ, O'Hare MJ, Arcaro A (2008). Immortalization of human alveolar epithelial cells to investigate nanoparticle uptake. Am J Respir Cell Mol Biol.

[9] Li C, Liu H, Sun Y, Wang H, Guo F, Rao S (2009). PAMAM nanoparticles promote acute lung injury by inducing autophagic cell death through the Akt-TSC2-mTOR signaling pathway. J Mol Cell Biol.

[10] Song Y, Li X, Du X (2009). Exposure to nanoparticles is related to pleural effusion, pulmonary fibrosis and granuloma. Eur Respir J.

[11] Adamcakova-Dodd A, Stebounova LV, Kim JS, Vorrink SU, Ault AP, O'Shaughnessy PT (2014). Toxicity assessment of zinc oxide nanoparticles using sub-acute and sub-chronic murine inhalation models. Particle Fibre Toxicol.

[12] Liu M, Zhang H, Slutsky AS (2009). Acute lung injury: a yellow card for engineered nanoparticles?. J Mol Cell Biol.

[13] Sung JC, Pulliam BL, Edwards DA (2007). Nanoparticles for drug delivery to the lungs. Trends Biotechnol.

[14] Svenson S, Tomalia DA (2005). Dendrimers in biomedical applications–reflections on the field. Adv Drug Deliv Rev.

[15] Tomalia DA (1991). Dendrimer research. Science.

[16] Chauhan AS, Diwan PV, Jain NK, Tomalia DA (2009). Unexpected *in vivo* anti-inflammatory activity observed for simple, surface functionalized poly (amidoamine) dendrimers. Biomacromolecules.

[17] Kukowska-Latallo JF, Candido KA, Cao Z, Nigavekar SS, Majoros IJ, Thomas TP (2005). Nanoparticle targeting of anticancer drug improves therapeutic response in animal model of human epithelial cancer. Cancer Res.

[18] Zhou J, Neff CP, Liu X, Zhang J, Li H, Smith DD (2011). Systemic administration of combinatorial dsiRNAs via nanoparticles efficiently suppresses HIV-1 infection in humanized mice. Mol Ther.

[19] Nasr M, Najlah M, D'Emanuele A, Elhissi A (2014). PAMAM dendrimers as aerosol drug nanocarriers for pulmonary delivery via nebulization. Int J Pharm.

[20] Bai S, Thomas C, Ahsan F (2007). Dendrimers as a carrier for pulmonary delivery of enoxaparin, a low-molecular weight heparin. J Pharm Sci.

[21] Kukowska-Latallo JF, Raczka E, Quintana A, Chen C, Rymaszewski M, Baker JR (2000). Intravascular and endobronchial DNA delivery to murine lung tissue using a novel, nonviral vector. Hum Gene Ther.

[22] Nigavekar SS, Sung LY, Llanes M, El-Jawahri A, Lawrence TS, Becker CW (2004). 3H dendrimer nanoparticle organ/tumor distribution. Pharm Res.

[23] Duncan R, Izzo L (2005). Dendrimer biocompatibility and toxicity. Adv Drug Deliv Rev.

[24] Greish K, Thiagarajan G, Herd H, Price R, Bauer H, Hubbard D (2012). Size and surface charge significantly influence the toxicity of silica and dendritic nanoparticles. Nanotoxicology.

[25] Thiagarajan G, Greish K, Ghandehari H (2013). Charge affects the oral toxicity of poly(amidoamine) dendrimers. Eur J Pharm Biopharm.

[26] Jones CF, Campbell RA, Brooks AE, Assemi S, Tadjiki S, Thiagarajan G (2012). Cationic PAMAM dendrimers aggressively initiate blood clot formation. ACS Nano.

[27] Jones CF, Campbell RA, Franks Z, Gibson CC, Thiagarajan G, Vieira-de-Abreu A (2012). Cationic PAMAM dendrimers disrupt key platelet functions. Mol Pharm.

[28] Goldberg DS, Vijayalakshmi N, Swaan PW, Ghandehari H (2011). G3.5 PAMAM dendrimers enhance transepithelial transport of SN38 while minimizing gastrointestinal toxicity. J Control Release.

[29] Kolhatkar RB, Kitchens KM, Swaan PW, Ghandehari H (2007). Surface acetylation of polyamidoamine (PAMAM) dendrimers decreases cytotoxicity while maintaining membrane permeability. Bioconjug Chem.

[30] Ingenito EP, Mark L, Davison B (1994). Effects of acute lung injury on dynamic tissue properties. J Appl Physiol.

[31] Stimler NP, Hugli TE, Bloor CM (1980). Pulmonary injury induced by C3a and C5a anaphylatoxins. Am J Pathol.

[32] Sugerman HJ, Tatum JL, Strash AM, Hirsch JI, Greenfield LJ (1981). Effects of pulmonary vascular recruitment on gamma scintigraphy and pulmonary capillary protein leak. Surgery.

[33] Imai Y, Kuba K, Rao S, Huan Y, Guo F, Guan B (2005). Angiotensin-converting enzyme 2 protects from severe acute lung failure. Nature.

[34] Kuba K, Imai Y, Rao S, Gao H, Guo F, Guan B (2005). A crucial role of angiotensin converting enzyme 2 (ACE2) in SARS coronavirus-induced lung injury. Nat Med.

[35] Zou Z, Yan Y, Shu Y, Gao R, Sun Y, Li X (2014). Angiotensin-converting enzyme 2 protects from lethal avian influenza A H5N1 infections. Nat Commun.

[36] Crackower MA, Sarao R, Oudit GY, Yagil C, Kozieradzki I, Scanga SE (2002). Angiotensin-converting enzyme 2 is an essential regulator of heart function. Nature.

[37] Huang F, Guo J, Zou Z, Liu J, Cao B, Zhang S (2014). Angiotensin II plasma levels are linked to disease severity and predict fatal outcomes in H7N9-infected patients. Nat Commun.

[38] Wallace KB, Bailie MD, Hook JB (1978). Angiotensin-converting enzyme in developing lung and kidney. Am J Physiol.

[39] Liu HL, Zhang YL, Yang N, Zhang YX, Liu XQ, Li CG (2011). A functionalized single-walled carbon nanotube-induced autophagic cell death in human lung cells through Akt-TSC2-mTOR signaling. Cell Death Disease.

[40] Sun T, Yan Y, Zhao Y, Guo F, Jiang C (2012). Copper oxide nanoparticles induce autophagic cell death in A549 cells. PLoS One.

[41] Yu KY, Wang YP, Wang LH, Jian Y, Zhao XD, Chen JW (2014). Mitochondrial KATP channel involvement in angiotensin II-induced autophagy in vascular smooth muscle cells. Basic Res Cardiol.

[42] Yang P, Gu H, Zhao Z, Wang W, Cao B, Lai C (2014). Angiotensin-converting enzyme 2 (ACE2) mediates influenza H7N9 virus-induced acute lung injury. Scientific Rep.

[43] Taylor E, Webster TJ (2011). Reducing infections through nanotechnology and nanoparticles. Int J Nanomedicine.

[44] Hoet P, Legiest B, Geys J, Nemery B (2009). Do nanomedicines require novel safety assessments to ensure their safety for long-term human use?. Drug Saf.

[45] Chappell MC, Millsted A, Diz DI, Brosnihan KB, Ferrario CM (1991). Evidence for an intrinsic angiotensin system in the canine pancreas. J Hypertens.

