# Supplementary material for: Cationic nanoparticles directly bind angiotensin-converting enzyme 2 and induce acute lung injury in mice
Source: Part Fibre Toxicol. 2015 Mar 7;12:4. doi: 10.1186/s12989-015-0080-x (PMC4395934; doi:10.1186/s12989-015-0080-x)
Supplement: Additional file 1: Table S1. — Physical-chemical characteristics of PAMAMs used in this study. [file 12989_2015_80_MOESM1_ESM.pdf]

Generation	Molecular weight	Core composition	Surface group	Number of surface groups	Charge	Size (nm)	CAS number
Generation 1.0	1429.85	ethylenediamine core	amino	8	positive	2.2	142986-44-5
Generation 2.0	3256.18	ethylenediamine core	amino	16	positive	2.9	93376-66-0
Generation 3.0	6908.84	ethylenediamine core	amino	32	positive	3.6	153891-46-4
Generation 4.0	14214.17	ethylenediamine core	amino	64	positive	4.5	163442-67-9
Generation 5.0	28824.81	ethylenediamine core	amino	128	positive	5.4	163442-68-0
Generation 6.0	58046.11	ethylenediamine core	amino	256	positive	6.7	163442-69-1
Generation 7.0	116488.71	ethylenediamine core	amino	512	positive	8.1	163442-70-4
Generation 3.5	12927.69	ethylenediamine core	carboxylate	64	negative	-	192948-77-9
Generation 4.5	26251.86	ethylenediamine core	carboxylate	128	negative	-	202009-66-3
Generation 5.5	52900.21	ethylenediamine core	carboxylate	256	negative	-	202009-67-4
Generation 7.5	212790.3	ethylenediamine core	carboxylate	1024	negative	-	-

(–) not available

Table S1 Physical-chemical characteristics of PAMAMs used in this study.
