# Supplementary material for: Cationic nanoparticles directly bind angiotensin-converting enzyme 2 and induce acute lung injury in mice
Source: Part Fibre Toxicol. 2015 Mar 7;12:4. doi: 10.1186/s12989-015-0080-x (PMC4395934; doi:10.1186/s12989-015-0080-x)
Supplement: Additional file 2: Figure S1. — Effects of various nanomaterials on ACE2 expression and plasma angiotensin II levels. (A)Plasma AngII levels of mice receiving the vehicle (control) and mice instilled with the indicated nanoparticles (all used at15 μg/g) at 3 hrs after challenge. n = 4 mice per group. (B) ACE and ACE2 protein levels in lungs 3 hrs after the instillation of t he indicated nanoparticles (15 μg/g). The upper panels show representative Western blots. The bar graphs show ACE and ACE2 levels as ratios to β-actin. n = 3 mice per group. Data are shown as the mean values ± SEM. For A and B, no differences were detected between the nanoparticle-treated groups and the vehicle control groups using two-tailed t-tests. [file 12989_2015_80_MOESM2_ESM.pdf]

Supplementary Material

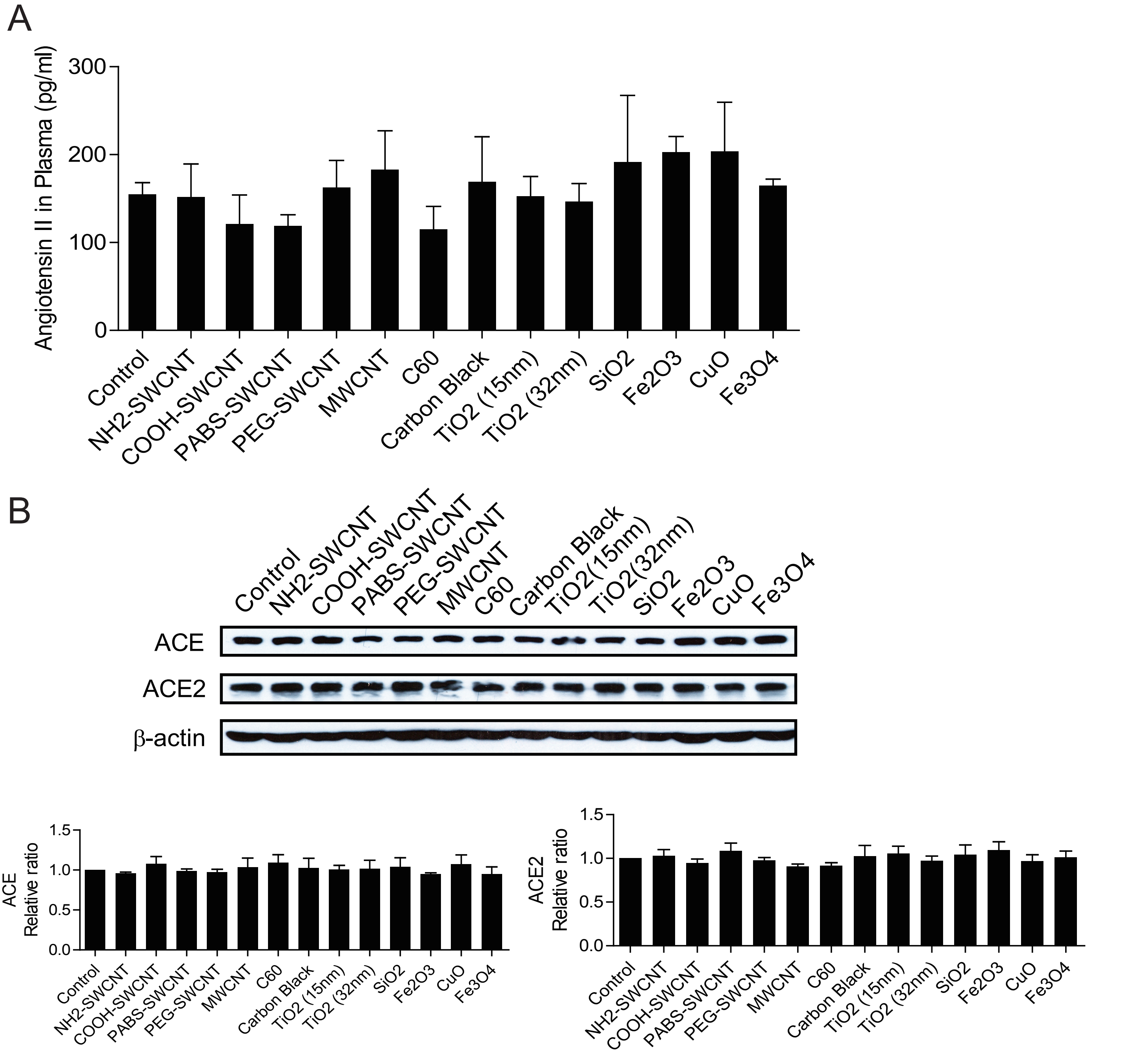

Figure S1. Effects of various nanomaterials on ACE2 expression and plasma angiotensin II levels. (A)Plasma AngII levels of mice receiving the vehicle (control) and mice instilled with the indicated nanoparticles (all used at15 μg/g) at 3 hrs after challenge. n = 4 mice per group. (B) ACE and ACE2 protein levels in lungs 3 hrs after the instillation of t he indicated nanoparticles (15 μg/g). The upper panels show representative Western blots. The bar graphs show ACE and ACE2 levels as ratios to β-actin. n = 3 mice per group. Data are shown as the mean values ±SEM. For A and B, no differences were detected between the nanoparticle -treated groups and the vehicle control groups using two-tailed t-tests.
