# Supplementary material for: Cationic nanoparticles directly bind angiotensin-converting enzyme 2 and induce acute lung injury in mice
Source: Part Fibre Toxicol. 2015 Mar 7;12:4. doi: 10.1186/s12989-015-0080-x (PMC4395934; doi:10.1186/s12989-015-0080-x)
Supplement: Additional file 3: Figure S2. — SPR signal of cationic polyamidoamine dendrimer nanoparticles combined with ACE2. The binding abilities of G2, G3, G4, G6 and G7 nanoparticles with recombinant ACE2 at different concentrations were measured by surface plasmon resonance (SPR). The detailed dynamic binding constants and equilibrium dissociation constants are shown in Additional file 1: Table S1. [file 12989_2015_80_MOESM3_ESM.pdf]

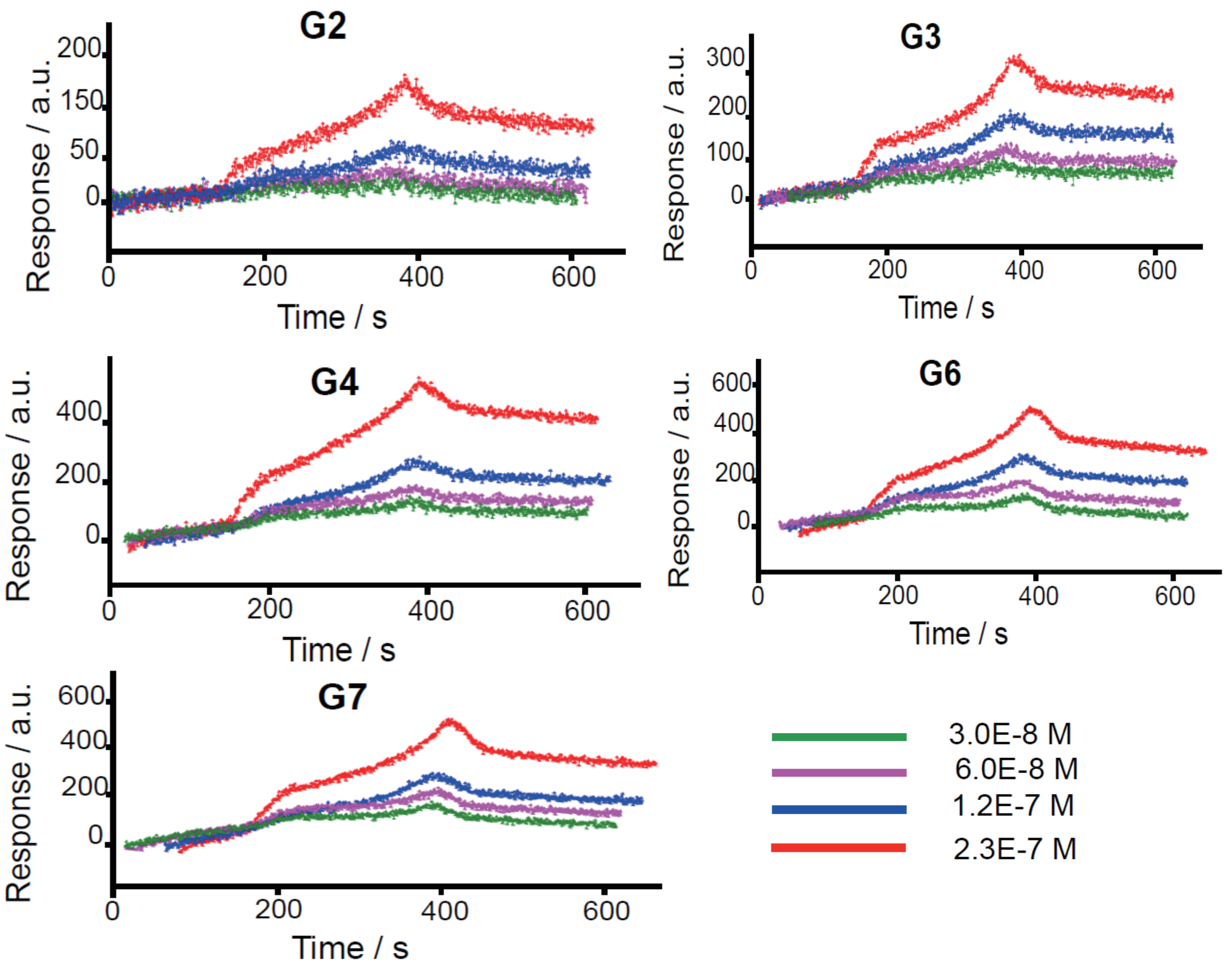

Figure S2. SPR signal of cationic polyamidoamine dendrimer nanoparticles combined with ACE2.The binding abilities of G2, G3, G4, G6 and G7 nanoparticles with recombinant ACE2 at different concentrations were measured by surface plasmon resonance (SPR). The detailed dynamic binding constants and equilibrium dissociation constants are shown in Supplementary Table S1.
