# Supplementary material for: Cationic nanoparticles directly bind angiotensin-converting enzyme 2 and induce acute lung injury in mice
Source: Part Fibre Toxicol. 2015 Mar 7;12:4. doi: 10.1186/s12989-015-0080-x (PMC4395934; doi:10.1186/s12989-015-0080-x)
Supplement: Additional file 4: Table S2. — Kinetic parameters for the binding between cationic polyamidoamine dendrimer nanoparticles and ACE2. Binding of cationic polyamidoamine dendrimer nanoparticles, including the cationic polymers G1, G2, G3, G4, G5, G6, and G7 and the anionicpolymers G3.5, G4.5, G5.5, and G7.5, to recombinant ACE2 was assessed using SPR. The detailed data for the ka (dynamic binding constant), kd (dynamic dissociation constant), KA (equilibrium binding constant) and KD (equilibrium dissociation constant) are indicated. N.D. indicates that no SPR signal was detected. [file 12989_2015_80_MOESM4_ESM.pdf]

	ka(1/Ms)	kd(1/s)	KA(1/M)	KD(M)
G1	N.D.			
G2	65.8	7.23E-07	9.1E+0.7	1.10E-08
G3	114	1.2E-06	9.58E+0.7	1.04E-08
G4	75.8	4.83E-07	1.57E+08	6.37E-09
G5	3.56E+04	1.09E-06	3.28E+10	3.05E-11
G6	153	2.25E-06	6.78+07	1.48E-08
G7	190	9.66E-07	1.97E+08	5.07E-0.9
G3.5	N.D.			
G4.5	N.D.			
G5.5	N.D.			
G7.5	N.D.			

Table S2. Kinetic parameters for the binding between cationic polyamidoamine dendrimer nanoparticles and ACE2. Binding of cationic polyamidoamine dendrimer nanoparticles, including the cationic polymers G1, G2, G3, G4, G5, G6, and G7 and the anionicpolymers G3.5, G4.5, G5.5, and G7.5, to recombinant ACE2 was assessed using SPR. The detailed data for the ka (dynamic binding constant), kd (dynamic dissociation constant), KA (equilibrium binding constant) and KD (equilibrium dissociation constant) are indicated. N.D. indicates that no SPR signal was detected.
